# Supplementary material for: Diversification of DNA-Binding Specificity by Permissive and Specificity-Switching Mutations in the ParB/Noc Protein Family
Source: Cell Rep. 2020 Jul 21;32(3):107928. doi: 10.1016/j.celrep.2020.107928 (PMC7383237; doi:10.1016/j.celrep.2020.107928)
Supplement: Document S1. Figures S1–S7 and Tables S1–S5 [file mmc1.pdf]

Cell Reports, Volume *32*

Supplemental Information

Diversification of DNA-Binding Specificity by Permissive and Specificity-Switching Mutations in the ParB/Noc Protein Family

Adam S.B. Jalal, Ngat T. Tran, Clare E. Stevenson, Elliot W. Chan, Rebecca Lo, Xiao Tan, Agnes Noy, David M. Lawson, and Tung B.K. Le

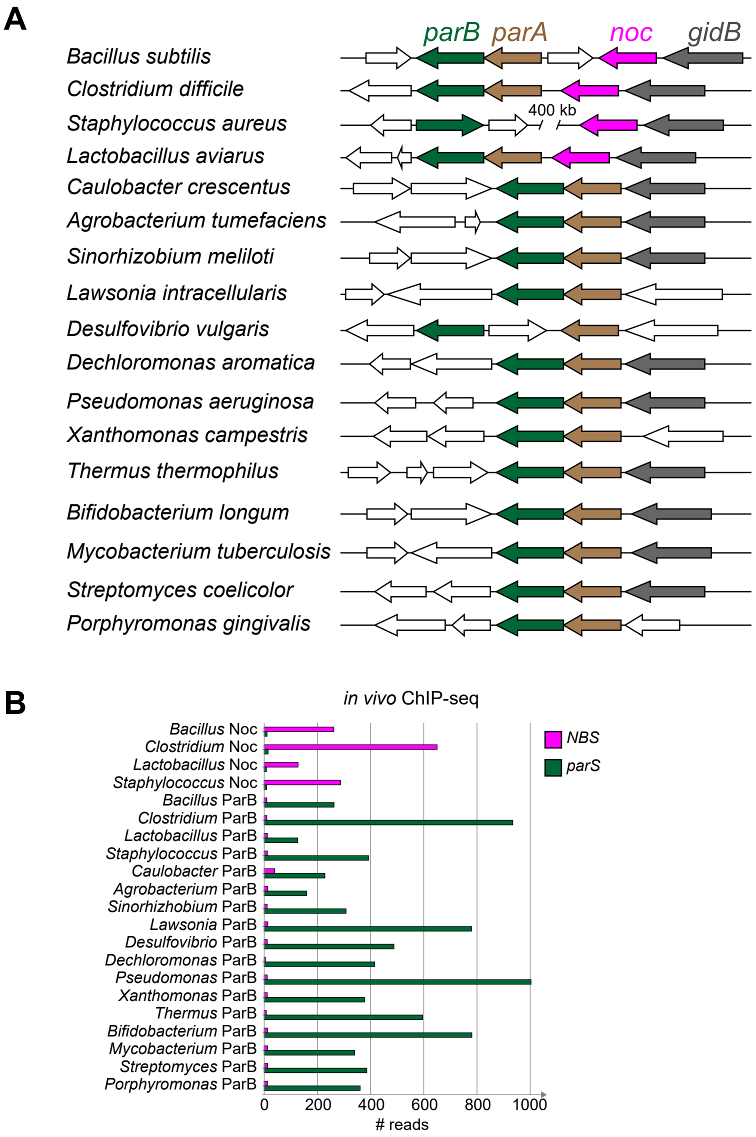

Figure S1. DNA-binding specificity for *parS* and *NBS* is conserved among ParB and Noc orthologs. Related to Figure 1. (A) Genomic context of ParB- and Noc-encoding genes in various bacterial species. *parB*, *parA*, *noc*, and the highly conserved *gidB* gene, are colored in dark green, brown, magenta, and grey, respectively. Genes at the border of the *parB-parA-noc* cluster (open arrows) vary between bacterial species. **(B)** The *in vivo* binding preferences of ParB/Noc to *parS/NBS* as measured by ChIP-seq. An *E. coli* strain with a single *parS* and *NBS* site engineered onto the chromosome was used as a heterologous host for expression of FLAG-tagged ParB/Noc. For ChIP-seq data, reads in a 100-bp window surrounding the *parS/NBS* site were quantified and used as a proxy for the enrichment of immunoprecipitated *parS* or *NBS* DNA.

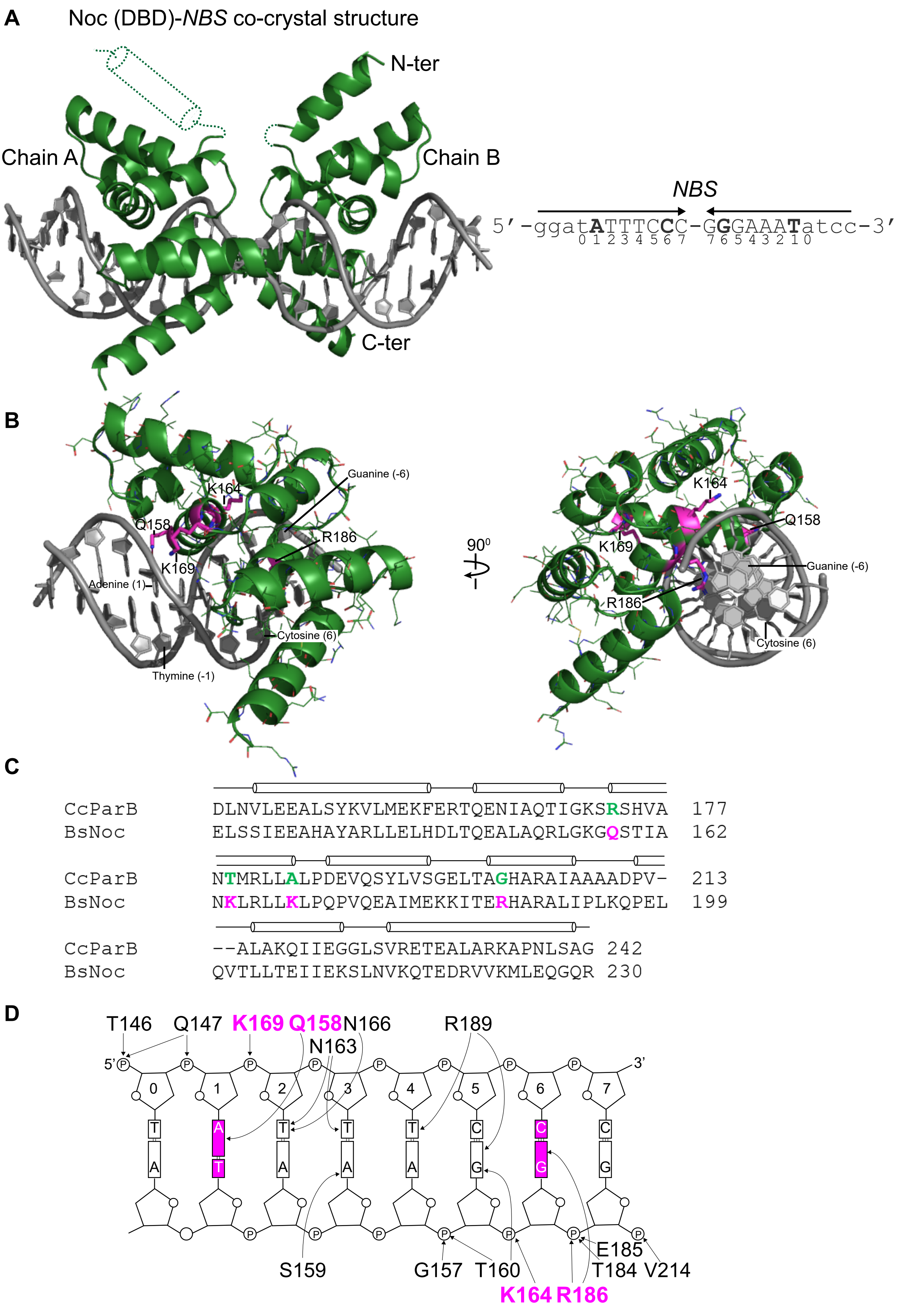

figure legend on the next page

Figure S2. Co-crystal structures of the Noc (DBD)-*NBS* complex. Related to Figure 4. **(A)** The structure of two Noc (DNA-binding domain) monomers (dark green) in complex with a 22-bp *NBS* DNA (grey). A helix (residues 113-125, dotted dark green cylinder) in chain A is not resolved. The nucleotide sequence of the 22-bp *NBS* site is shown on the left-hand side; bases (Adenine 1 and Cytosine 6) that are different from *parS* are in bold. **(B)** One monomer of Noc (DBD) is shown in complex with an *NBS* half-site; four core specificity residues are shown in stick presentation, labeled, and colored in magenta. Other residues surrounding specificity residues are showed as lines and colored in dark green. **(C)** Amino acid sequences of *C. crescentus* ParB and *B. subtilis* Noc with the positions of four specificity residues highlighted in dark green and magenta, respectively. Secondary structures are shown above the sequence alignment. **(D)** Schematic representation of Noc (DBD)-*NBS* interactions. For simplicity, only half of *NBS* is shown. The two bases at position 1 and 6 that are different between *parS* and *NBS* are highlighted in magenta. The four core specificity residues are also colored in magenta.

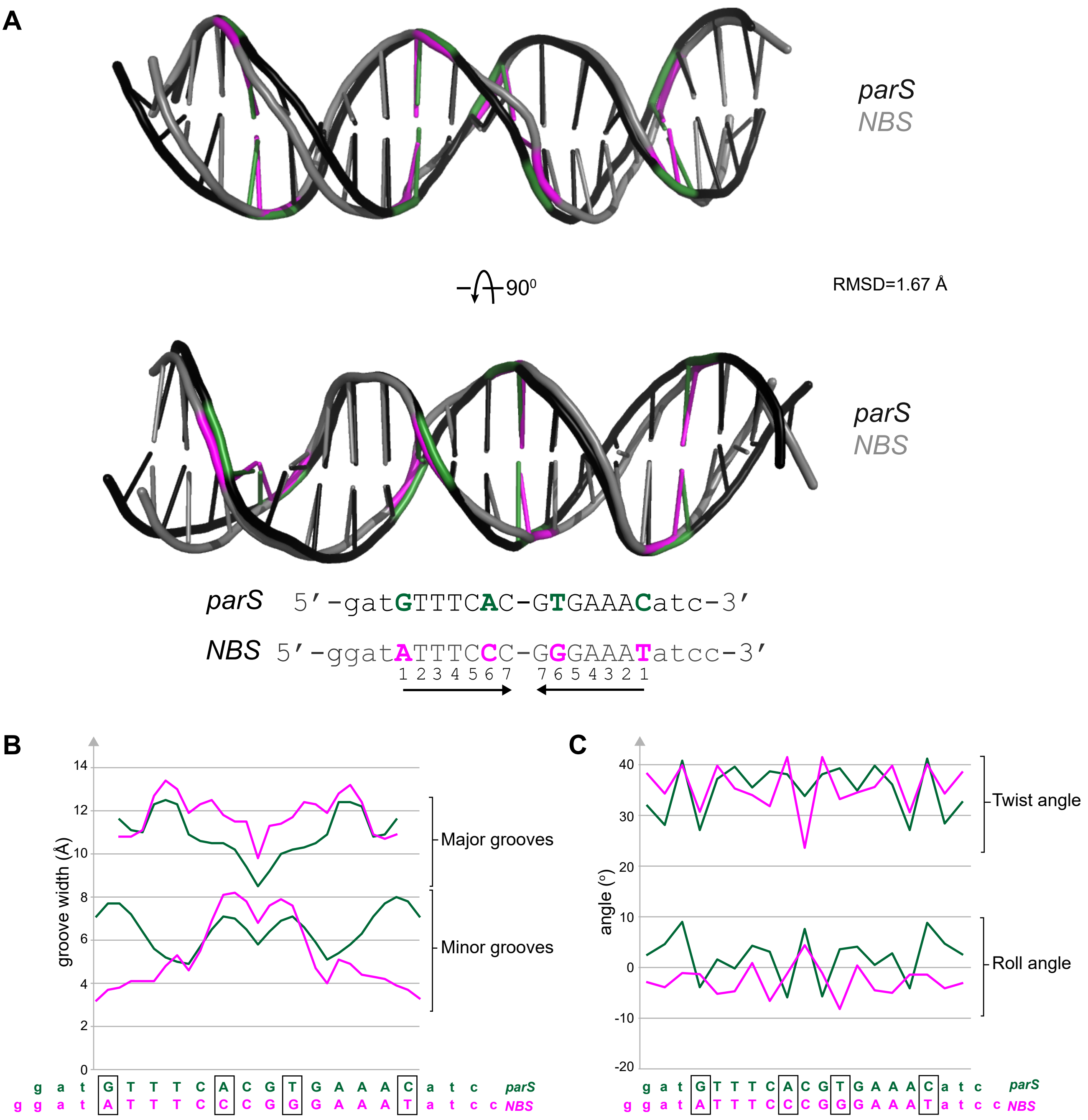

Figure S3. Conformational changes at *parS* and *NBS* DNA within the two co-crystal structures. Related to Figure 4. (A) Superimposition of *parS* and *NBS* DNA structures, root-mean-square deviation (RMSD) value is also shown. Bases that differ between *parS* (dark green) and *NBS* (magenta) are highlighted. **(B)** The major and minor groove widths of the bound DNA (*parS*: dark green, *NBS*: magenta). **(C)** The roll and twist angles for each base pair step of the bound DNA (*parS*: dark green, *NBS*: magenta).

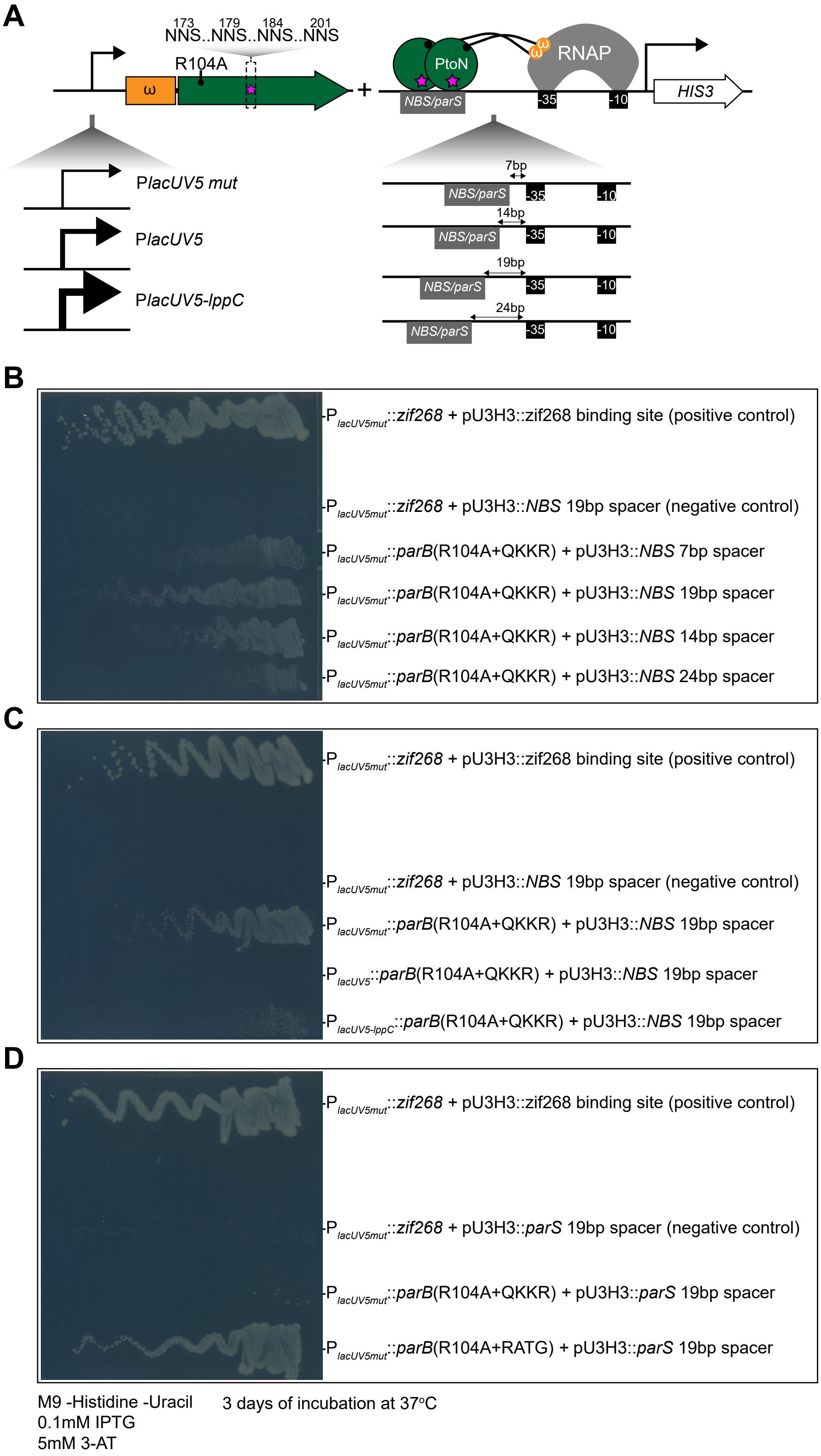

Figure S4. Optimization of bacterial one-hybrid (B1H) assay to select for variants that bind *parS* or *NBS*. Related to Figure 5. (A) The strength of the promoter that drives the expression of *parB* variants and the distances between the *parS* or *NBS* binding site to the core -10 -35 promoter were optimized. **(B)** A 19-bp gap between *NBS/parS* and the core promoter is optimal, based on the streak test for cell growth in a minimal medium lacking histidine. **(C)** A weak promoter ($P_{lacUV5mut}$) is optimal, based on the streak test for cell growth in a minimal medium lacking histidine. **(B and D)** The presence of *parS* or *NBS* upstream of HIS3, in the absence of ParB variants, did not auto-activate its expression.

A

	number of quality-filter reads
Pre-selection, replicate 1	6,954,115
Pre-selection, replicate 2	8,623,273
Pre-selection, replicate 3	7,201,395
Total	22,778,783
Post-selection NBS, replicate 1	6,294,658
Post-selection NBS, replicate 2	7,487,037
Post-selection NBS, replicate 3	6,689,637
Total	20,471,332
Post-selection parS, replicate 1	5,380,569
Post-selection parS, replicate 2	8,572,521
Post-selection parS, replicate 3	7,269,057
Total	21,222,147

B

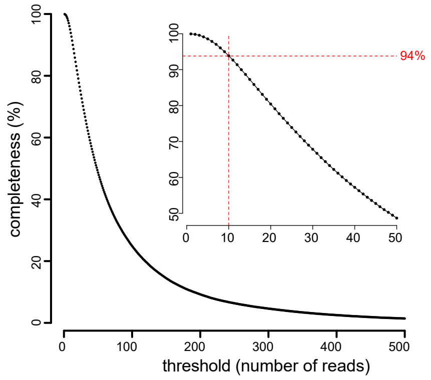

C

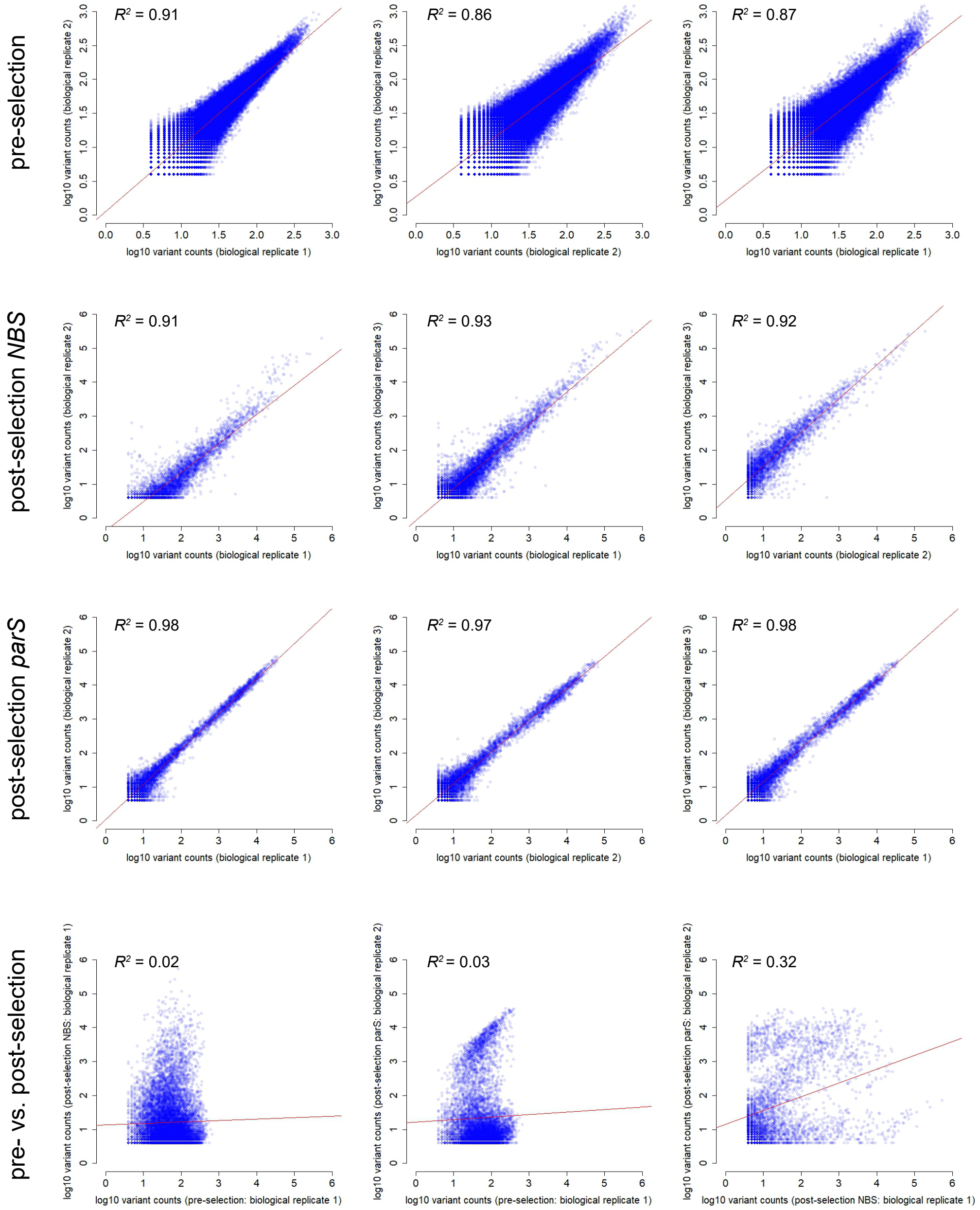

Figure S5. Statistics of deep sequencing reads and completeness of starting libraries. Related to Figure 5. (A) Number of quality-filtered reads for each biological replicate, for pre- and post-selection libraries. **(B)** The completeness of pre-selection libraries at different thresholds. A completeness of 100% means that all 160,000 variants lacking stop codons were present in the pre-selection library. In the starting library, greater than 94% of the predicted variants were represented by at least 10 reads. **(C)** Reproducibility of biological replicates: pre- vs. pre-selection replicates and pre- vs. post-selection replicates. Pearson's correlation coefficients (R^2) are also shown. Red lines show least squares best fits.

A

pU3H3::*zif268* (negative control) pU3H3::*parS* 19bp spacer pU3H3::*NBS* 19bp spacer $P_{lacUV5mut}$::*parB**

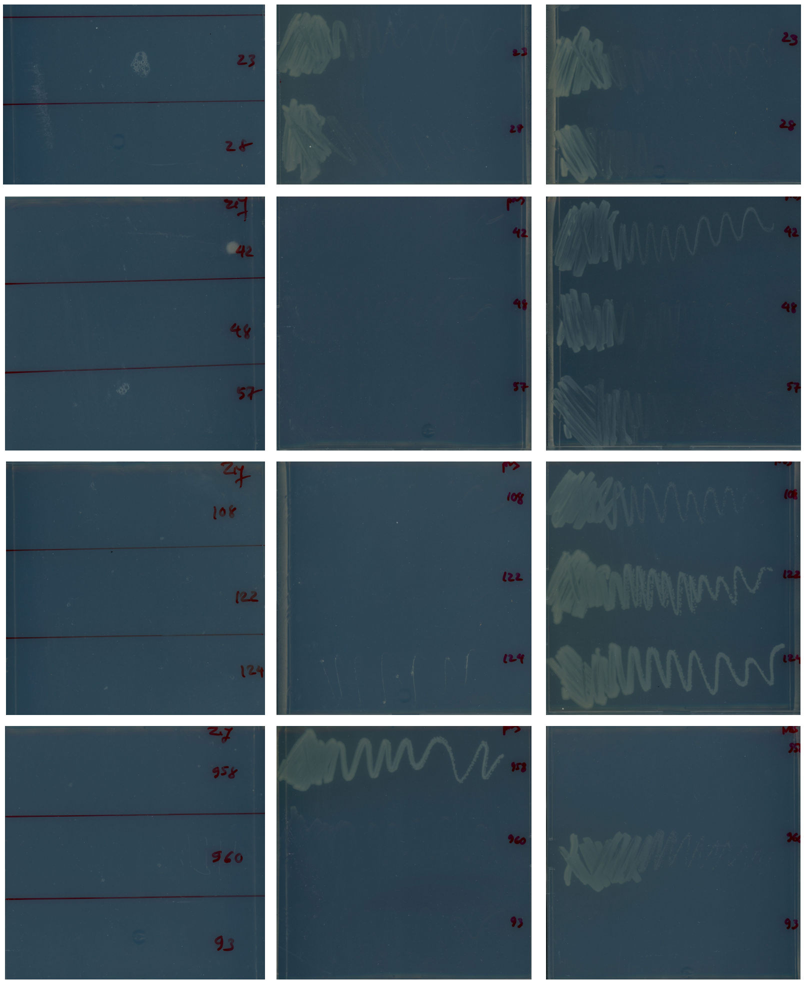

M9 -Histidine -Uracil 3 days of incubation at 37°C
0.1mM IPTG
5mM 3-AT

B

$P_{lacUV5mut}$::*parB**	K_D^{parS} (nM)	K_D^{NBS} (nM)
RRMT	12 ± 2.4	45 ± 11
RRLT	7.0 ± 3.0	26 ± 9.0
QRMR	no binding	60 ± 26
QSRR	no binding	30 ± 16
QTNR	no binding	800 ± 92
QRYR	no binding	65 ± 12
QRRR	no binding	4.0 ± 1.2
QRKR	no binding	4.0 ± 3.0
WT (RTAG)	11 ± 7.0	no binding
QKKR	no binding	28 ± 5.0
TEPG	no binding	no binding

Figure S6. Validation of selected variants from the deep mutational scanning experiments. Related to Figure 5. (A) Validation by pairwise bacterial one-hybrid assays. The ability of nine selected variants to grow on a minimal medium lacking histidine (but supplemented with 5mM 3-AT to increase the stringency) was assessed by a streak test. Plasmid harboring a binding site of an eukaryotic transcription factor (*zif268*) served as a negative control. **(B)** Validation by bio-layer interferometry assays. Selected variants were expressed, purified, and K_D ± SD were measured by bio-layer interferometry assay.

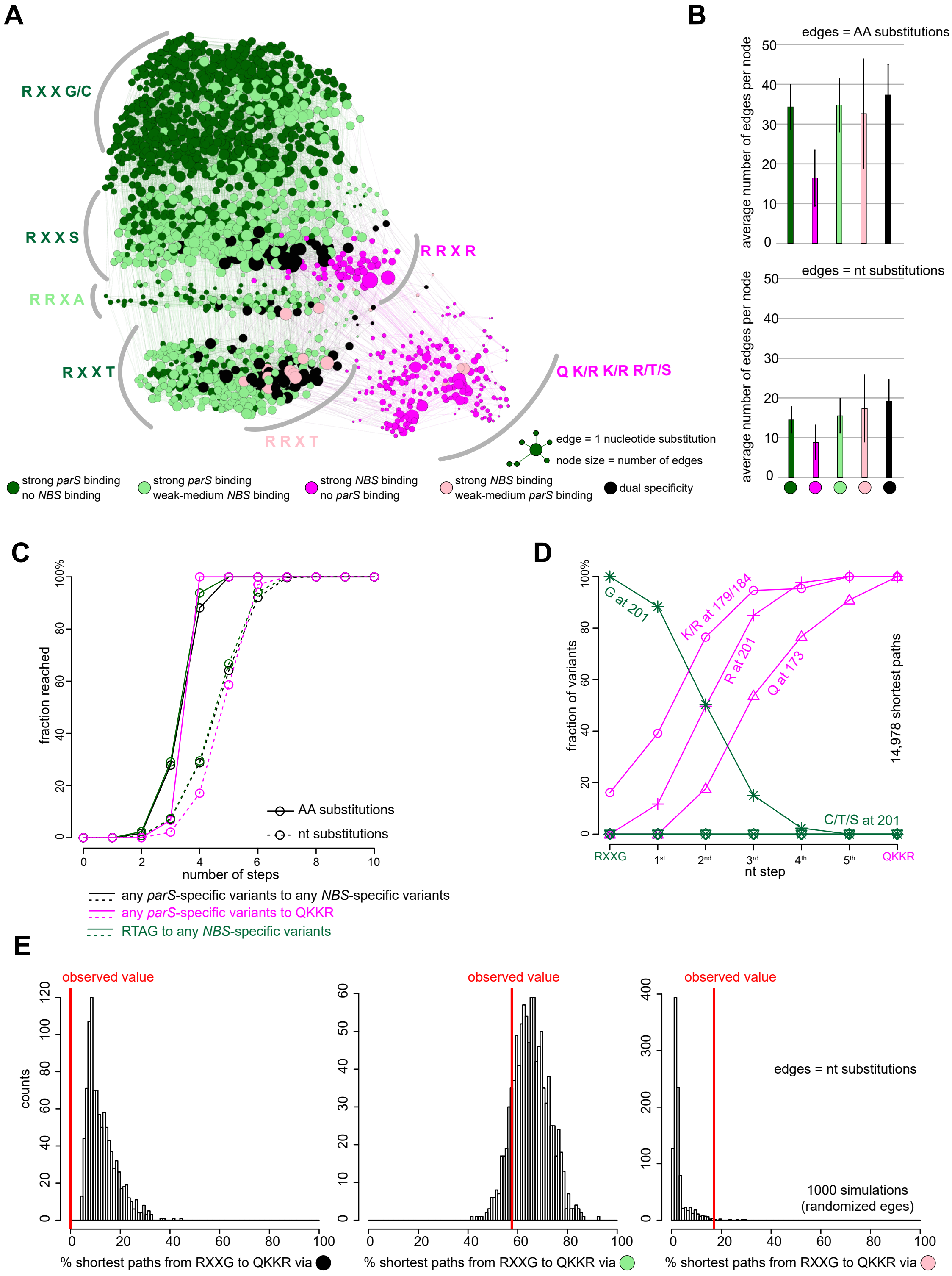

Figure S7. Deep mutational scanning experiments reveal the common properties of mutational paths. Related to Figure 6. (A) A force-directed network graph connecting strong *parS*-binding variants to strong *NBS*-binding variants. Nodes represent individual variants, and edges represent single nucleotide (nt) substitutions. Node sizes are proportional to their corresponding numbers of edges. Node colors correspond to different classes of variants. **(B)** Average number of edges per node. **(C)** Cumulative fraction of variants that reached their destinations in a given number of amino acid (solid line) or nucleotide (dotted line) substitutions. Black lines: from any *parS*-specific variants to any *NBS*-specific variants. Magenta lines: from any *parS*-specific variants to QKKR. Dark green lines: from RTAG to any *NBS*-specific variants. **(D)** Fraction of intermediates on all shortest paths from highly *parS*-specific RXXG variants to the *NBS*-preferred QKKR that have permissive amino acids (K/R) at either position 179/184 or both, or have R at position 201, or Q at position 173, or C/T/S at position 201 after a given number of nt steps. **(E)** Percentage of shortest paths that traversed black, light green, or pink variants to reach QKKR from any of the highly *parS*-specific RXXG variants (red lines). The result was compared to ones from 1,000 simulations where the by-nt-substitution edges were shuffled randomly while keeping the total number of nodes, edges, and graph density constant.

TABLE S1. STRAINS. Related to STAR Methods.

Strains	Strains/descriptions	Source
AB1157	*thr-1, ara-14, leuB6, Δ(gpt-proA)62, lacY1, tsx-33, supE44, galK2, rac-, hisG4(Oc), rfbD1, mgl-51, rpsL31, kdgK51, xyl-5, mtl-1, argE3 (Oc), thi-1, qsr-*	Yale *E. coli* Genetic Stock Center
DH5α	*E. coli* host for DNA cloning and propagation of plasmid	Le lab collection
Rosetta (DE3)	*E. coli* host for protein overexpression from an IPTG-inducible T7 promoter	Merck
CJW4025	BL21 pET21b::*parB-(his)6*	Gift from Christine Jacob-Wagner (Lim et al., 2014)
TLE3000	*AB1157 ybbD::parS::markerless ygcE::NBS::markerless*	This study
TLE3001	*USO rpoZ- hisB- pyrF-*	Scott Wolfe (Noyes et al., 2008) via Addgene
	TLE3000+pUT18C::1xFLAG-*Bacillus subtilis* Noc	This study
	TLE3000+pUT18C::1xFLAG-*Clostridium difficile* Noc	This study
	TLE3000+pUT18C::1xFLAG-*Lactobacillus aviarius* Noc	This study
	TLE3000+pUT18C::1xFLAG-*Staphylococcus aureus* Noc	This study
	TLE3000+pUT18C::1xFLAG-*Bacillus subtilis* ParB	This study
	TLE3000+pUT18C::1xFLAG-*Clostridium difficile* ParB	This study
	TLE3000+pUT18C::1xFLAG-*Lactobacillus aviarius* ParB	This study
	TLE3000+pUT18C::1xFLAG-*Staphylococcus aureus* ParB	This study
	TLE3000+pUT18C::1xFLAG-*Caulobacter crescentus* ParB	This study
	TLE3000+pUT18C::1xFLAG-*Agrobacterium tumefaciens* ParB	This study
	TLE3000+pUT18C::1xFLAG-*Sinorhizobium meliloti* ParB	This study
	TLE3000+pUT18C::1xFLAG-*Lawsonia intracellularis* ParB	This study
	TLE3000+pUT18C::1xFLAG-*Desulfovibrio vulgaris* ParB	This study
	TLE3000+pUT18C::1xFLAG-*Dechloromonas aromatica* ParB	This study
	TLE3000+pUT18C::1xFLAG-*Pseudomonas aeruginosa* ParB	This study
	TLE3000+pUT18C::1xFLAG-*Xanthomonas campestris* ParB	This study
	TLE3000+pUT18C::1xFLAG-*Thermus thermophilus* ParB	This study
	TLE3000+pUT18C::1xFLAG-*Bifidobacterium longum* ParB	This study
	TLE3000+pUT18C::1xFLAG-*Mycobacterium tuberculosis* ParB	This study
	TLE3000+pUT18C::1xFLAG-*Streptomyces coelicolor* ParB	This study
	TLE3000+pUT18C::1xFLAG-*Porphyromonas gingivalis* ParB	This study
	TLE3001 + pB1H2-w2::*zif268* + pU3H3::*zif268* binding site	This study
	TLE3001 + pB1H2-w2::*zif268* + pU3H3::19bp-*NBS*	This study
	TLE3001 + pB1H2-w2::*Caulobacter* ParB (R104A + Q173K179K184R201) + pU3H3::7bp-*NBS*	This study
	TLE3001 + pB1H2-w2::*Caulobacter* ParB (R104A + Q173K179K184R201) + pU3H3::14bp-*NBS*	This study
	TLE3001 + pB1H2-w2::*Caulobacter* ParB (R104A + Q173K179K184R201) + pU3H3::19bp-*NBS*	This study
	TLE3001 + pB1H2-w2::*Caulobacter* ParB (R104A + Q173K179K184R201) + pU3H3::24bp-*NBS*	This study
	TLE3001 + pB1H2-w5::*Caulobacter* ParB (R104A +	This study

	Q173K179K184R201) + pU3H3::19bp-*NBS*	
	TLE3001 + pB1H2-w5L::*Caulobacter* ParB (R104A + Q173K179K184R201) + pU3H3::19bp-*NBS*	This study
	TLE3001 + pB1H2-w2::*zif268* + pU3H3::19bp-*parS*	This study
	TLE3001 + pB1H2-w2::*Caulobacter* ParB (R104A + Q173K179K184R201) + pU3H3::19bp-*parS*	This study
	TLE3001 + pB1H2-w2::*Caulobacter* ParB (R104A + R173A179T184G201) + pU3H3::19bp-*parS*	This study
	BL21 Rosetta pRARE + various pET21b-based protein overexpression vectors (see the plasmid list for the complete collection of protein overexpression plasmids)	This study

TABLE S2. PLASMIDS. Related to STAR Methods.

Plasmids	Description	Source
pENTR::D-TOPO	ENTRY vector for Gateway cloning, kanamycin^R	Invitrogen
	pENTR::*Bacillus subtilis* Noc	This study
	pENTR::*Clostridium difficile* Noc	This study
	pENTR::*Lactobacillus aviarius* Noc	This study
	pENTR::*Staphylococcus aureus* Noc	This study
	pENTR::*Bacillus subtilis* ParB	This study
	pENTR::*Clostridium difficile* ParB	This study
	pENTR::*Lactobacillus aviarius* ParB	This study
	pENTR::*Staphylococcus aureus* ParB	This study
	pENTR::*Caulobacter crescentus* ParB	This study
	pENTR::*Agrobacterium tumefaciens* ParB	This study
	pENTR::*Sinorhizobium meliloti* ParB	This study
	pENTR::*Lawsonia intracellularis* ParB	This study
	pENTR::*Desulfovibrio vulgaris* ParB	This study
	pENTR::*Dechloromonas aromatica* ParB	This study
	pENTR::*Pseudomonas aeruginosa* ParB	This study
	pENTR::*Xanthomonas campestris* ParB	This study
	pENTR::*Thermus thermophilus* ParB	This study
	pENTR::*Bifidobacterium longum* ParB	This study
	pENTR::*Mycobacterium tuberculosis* ParB	This study
	pENTR::*Streptomyces coelicolor* ParB	This study
	pENTR::*Porphyromonas gingivalis* ParB	This study
pML477	Gateway-cloning destination vector for fusion of protein interest to an N-terminally FLAG tag, xylose-inducible promoter, high-copy number plasmid, spectinomycin^R	Gift from Michael Laub
pET21b::ParB-(His)6	overexpression of ParB-(His)$_6$ from an IPTG-inducible T7 promoter	Promega
	pET21b::*Caulobacter crescentus*-ParB-(His)$_6$ (WT)	Gift of Christine Jacob Wagner (Lim et al., 2014)
	pET21b::*Caulobacter crescentus*-ParB-(His)$_6$ (DBD)	This study
	pET21b::*Bacillus subtilis*-Noc-(His)$_6$	This study
	pET21b::*Bacillus subtilis*-Noc-(His)$_6$ (DBD)	This study
	pET21b::PtoN1-(His)$_6$ (Q173T179A184G201)	This study
	pET21b::PtoN2-(His)$_6$ (R173K179A184G201)	This study
	pET21b::PtoN3-(His)$_6$ (R173T179K184G201)	This study
	pET21b::PtoN4-(His)$_6$ (R173T179A184R201)	This study
	pET21b::PtoN5-(His)$_6$ (Q173K179A184G201)	This study
	pET21b::PtoN6-(His)$_6$ (Q173T179K184G201)	This study
	pET21b::PtoN7-(His)$_6$ (Q173T179A184R201)	This study
	pET21b::PtoN8-(His)$_6$ (R173K179K184G201)	This study
	pET21b::PtoN9-(His)$_6$ (R173K179A184R201)	This study
	pET21b::PtoN10-(His)$_6$ (R173T179K184R201)	This study

	pET21b::PtoN11-(His)$_6$ (Q173K179K184G201)	This study
	pET21b::PtoN12-(His)$_6$ (Q173K179A184R201)	This study
	pET21b::PtoN13-(His)$_6$ (Q173T179K184R201)	This study
	pET21b::PtoN14-(His)$_6$ (R173K179K184R201)	This study
	pET21b::PtoN15-(His)$_6$ (Q173K179K184R201)	This study
	pET21b::*Caulobacter crescentus*-ParB-(His)$_6$ (Q162A)	This study
	pET21b::*Caulobacter crescentus*-ParB-(His)$_6$ (K171A)	This study
	pET21b::*Caulobacter crescentus*-ParB-(His)$_6$ (S172A)	This study
	pET21b::*Caulobacter crescentus*-ParB-(His)$_6$ (R173A)	This study
	pET21b::*Caulobacter crescentus*-ParB-(His)$_6$ (S174A)	This study
	pET21b::*Caulobacter crescentus*-ParB-(His)$_6$ (N178A)	This study
	pET21b::*Caulobacter crescentus*-ParB-(His)$_6$ (R181A)	This study
	pET21b::*Caulobacter crescentus*-ParB-(His)$_6$ (V226A)	This study
	pET21b::*Caulobacter crescentus*-ParB-(His)$_6$ (R227A)	This study
	pET21b::*Caulobacter crescentus*-ParB-(His)$_6$ (R234A)	This study
	pET21b::*Caulobacter crescentus*-ParB-(His)$_6$ (K245A)	This study
	pET21b::*Caulobacter crescentus*-ParB-(His)$_6$ (R248A)	This study
	pET21b::*Caulobacter crescentus*-ParB-(His)$_6$ (R204A)	This study
	pET21b::*Caulobacter crescentus*-ParB-(His)$_6$ (E230A)	This study
	pET21b::*Caulobacter crescentus*-ParB-(His)$_6$ (R251A)	This study
	pET21b::ParB-(His)$_6$ chimera 1	This study
	pET21b::ParB-(His)$_6$ chimera 4	This study
	pET21b::*Caulobacter crescentus*-ParB-(His)$_6$ (RRMT)	This study
	pET21b::*Caulobacter crescentus*-ParB-(His)$_6$ (RRLT)	This study
	pET21b::*Caulobacter crescentus*-ParB-(His)$_6$ (QRMR)	This study
	pET21b::*Caulobacter crescentus*-ParB-(His)$_6$ (QSRR)	This study
	pET21b::*Caulobacter crescentus*-ParB-(His)$_6$ (QTNR)	This study
	pET21b::*Caulobacter crescentus*-ParB-(His)$_6$ (QRYR)	This study
	pET21b::*Caulobacter crescentus*-ParB-(His)$_6$ (QRRR)	This study
	pET21b::*Caulobacter crescentus*-ParB-(His)$_6$ (QRKR)	This study
	pET21b::*Caulobacter crescentus*-ParB-(His)$_6$ (TEPG)	This study
pUT18C::1xFLAG-DEST	Destination vector for Gateway cloning, 1xFLAG tag fused to the N-terminus of protein of interest, carbenicillin^R	This study
	pUT18C::1xFLAG-*Bacillus subtilis* Noc	This study
	pUT18C::1xFLAG-*Clostridium difficile* Noc	This study
	pUT18C::1xFLAG-*Lactobacillus aviarius* Noc	This study
	pUT18C::1xFLAG-*Staphylococcus aureus* Noc	This study
	pUT18C::1xFLAG-*Bacillus subtilis* ParB	This study
	pUT18C::1xFLAG-*Clostridium difficile* ParB	This study
	pUT18C::1xFLAG-*Lactobacillus aviarius* ParB	This study
	pUT18C::1xFLAG-*Staphylococcus aureus* ParB	This study
	pUT18C::1xFLAG-*Caulobacter crescentus* ParB	This study
	pUT18C::1xFLAG-*Agrobacterium tumefaciens* ParB	This study
	pUT18C::1xFLAG-*Sinorhizobium meliloti* ParB	This study
	pUT18C::1xFLAG-*Lawsonia intracellularis* ParB	This study

	pUT18C::1xFLAG-*Desulfovibrio vulgaris* ParB	This study
	pUT18C::1xFLAG-*Dechloromonas aromatica* ParB	This study
	pUT18C::1xFLAG-*Pseudomonas aeruginosa* ParB	This study
	pUT18C::1xFLAG-*Xanthomonas campestris* ParB	This study
	pUT18C::1xFLAG-*Thermus thermophilus* ParB	This study
	pUT18C::1xFLAG-*Bifidobacterium longum* ParB	This study
	pUT18C::1xFLAG-*Mycobacterium tuberculosis* ParB	This study
	pUT18C::1xFLAG-*Streptomyces coelicolor* ParB	This study
	pUT18C::1xFLAG-*Porphyromonas gingivalis* ParB	This study
	pB1H2-w2::*zif268*	(Noyes et al., 2008) via Addgene
	pB1H2-w5::*zif268*	(Noyes et al., 2008) via Addgene
	pB1H2-w5L::*zif268*	(Noyes et al., 2008) via Addgene
	pU3H3::MCS	(Noyes et al., 2008) via Addgene
	pU3H3::*zif268* binding site	(Noyes et al., 2008) via Addgene
	pB1H2-w2::*Caulobacter* ParB (R104A + Q173K179K184R201)	This study
	pB1H2-w5::*Caulobacter* ParB (R104A + Q173K179K184R201)	This study
	pB1H2-w5L::*Caulobacter* ParB (R104A + Q173K179K184R201)	This study
	pB1H2-w2::*Caulobacter* ParB (R104A + R173A179T184G201)	This study
	pU3H3::7bp-*NBS*	This study
	pU3H3::14bp-*NBS*	This study
	pU3H3::19bp-*NBS*	This study
	pU3H3::24bp-*NBS*	This study
	pU3H3::19bp-*parS*	This study

TABLE S3. PRIMERS. Related to STAR Methods.

**Primers**	**Sequences**
	**For construction of pUT18C-1xFLAG-DEST**
1934	acaatttcacacaggaaacagctatggactacaaggacgacgacgacaagggctcg
1935	acttagttatatcgatgcatcgaaccactttgtacaagaaagctgaacgagaaac
1936	agctgtttcctgtgtgaaattgttatccgctcacaattc
1937	tcgatgcatcgatataactaagtaatatggtgcac
	**For ChIP-qPCR**
ybbD_parSF2	GTAAGATACCAGGGCAAGG
ybbD_parSR2	TTACTCTGCACAAGCATCA
ygcE_NBSF2	CGCTACGACGCGATGAATAA
ygcE_NBSR2	CTCTGGATCGAATCCACATTCC
ompG_F1	GCGGAGCCTTCAGTCTATTT
ompG_R1	CAAACCACGTTCCACGTTTAC
	**For bio-layer interferometry assays**
*NBS*_FOR	[Biotin]GGGAtaTTTCCCGGGAAAta
*NBS*_REV	taTTTCCCGGGAAAtaTCCC
*parS*_FOR	[Biotin]GGGAtgTTTCACGTGAAAca
*parS*_REV	tgTTTCACGTGAAAcaTCCC
site1_FOR	[Biotin]GGGAtgTTTCTCGAGAAAca
site1_REV	tgTTTCTCGAGAAAcaTCCC
site10_FOR	[Biotin]GGGAttTTTCgCGcGAAAaa
site10_REV	ttTTTCgCGcGAAAaaTCCC
site11_FOR	[Biotin]GGGAttTTTCcCGgGAAAaa
site11_REV	ttTTTCcCGgGAAAaaTCCC
site12_FOR	[Biotin]GGGAtaTTTCACGTGAAAta
site12_REV	taTTTCACGTGAAAtaTCCC
site13_FOR	[Biotin]GGGAtaTTTCtCGaGAAAta
site13_REV	taTTTCtCGaGAAAtaTCCC
site14_FOR	[Biotin]GGGAtaTTTCgCGcGAAAta
site14_REV	taTTTCgCGcGAAAtaTCCC
site2_FOR	[Biotin]GGGAtgTTTCGCGCGAAAca
site2_REV	tgTTTCGCGCGAAAcaTCCC
site3_FOR	[Biotin]GGGAtgTTTCCCGGGAAAca
site3_REV	tgTTTCCCGGGAAAcaTCCC
site4_FOR	[Biotin]GGGAtcTTTCACGTGAAAga
site4_REV	tcTTTCACGTGAAAgaTCCC
site5_FOR	[Biotin]GGGAtcTTTCtCGaGAAAga
site5_REV	tcTTTCtCGaGAAAgaTCCC
site6_FOR	[Biotin]GGGAtcTTTCgCGcGAAAga
site6_REV	tcTTTCgCGcGAAAgaTCCC
site7_FOR	[Biotin]GGGAtcTTTCcCGgGAAAga
site7_REV	tcTTTCcCGgGAAAgaTCCC
site8_FOR	[Biotin]GGGAttTTTCACGTGAAAaa
site8_REV	ttTTTCACGTGAAAaaTCCC
site9_FOR	[Biotin]GGGAttTTTCtCGaGAAAaa
site9_REV	ttTTTCtCGaGAAAaaTCCC
	**For the construction of pU3H3-*NBS* and pU3H3-*parS***
*NBS*_anneal_7bp_spacer_F	Ccgggtatttcccggaaataggg

*NBS*_anneal_7bp_spacer_R	aattccctatttcccgggaaatac
*NBS*_anneal_14bp_spacer_F	ccgggtatttcccgggaaataggcgcgccg
*NBS*_anneal_14bp_spacer_R	aattcggcgcgcctatttcccgggaaatac
*NBS*_anneal_19bp_spacer_F	ccgggtatttcccgggaaataggtttcgcgcgccg
*NBS*_anneal_19bp_spacer_R	Aattcggcgcgcgaaacctatttcccgggaaatac
*NBS*_anneal_24bp_spacer_F	ccgggtatttcccgggaaataggtttcttggcgcgcgccg
*NBS*_anneal_24bp_spacer_R	aattcggcgcgcgccaagaa acctatttcccgggaaatac
*parS*_anneal_19bp_spacer_F	ccgggtgtttcacgtgaaacaggtttcgcgcgccg
*parS*_anneal_19bp_spacer_R	ttaagccgcgcgctttggacaaagtgcactttgtg
	**For the construction of AB1157 *ybbD*::*parS* *ygcE*::*NBS* strain**
1940	aaatattggagctggattgcctgatgcttgtgcagagtaatgttccacgtggaacaattccggggatccgtcgacctg
1941	ttgacgacttcgatatgggatagactcttaattcaagcaatgtaggctggagctgcttcg
3139	ggggaatgtggattcgatccagagctggtcgaatgcgtaatatttcccgggaaataattccggggatccgtcgacctg
3140	tatgttcaggccgggcagtttcccgcccggccttcctcactgtaggctggagctgcttcgaag
	**For generation of Illumina libraries for deep mutational scanning experiments**
4nns_offset_0_F	ACACTCTTTCCCTACACGACGCTCTTCCGATCTgctcaaactattggcaagag
4nns_offset_1_F	ACACTCTTTCCCTACACGACGCTCTTCCGATCTtgctcaaactattggcaagag
4nns_offset_2_F	ACACTCTTTCCCTACACGACGCTCTTCCGATCTttgctcaaactattggcaagag
4nns_offset_3_F	ACACTCTTTCCCTACACGACGCTCTTCCGATCTattgctcaaactattggcaagag
4nns_offset_4_F	ACACTCTTTCCCTACACGACGCTCTTCCGATCTcattgctcaaactattggcaagag
4nns_R	GTGACTGGAGTTCAGACGTGTGCTCTTCCGATCTTTTTGCTAACGCTACGGGATC
NEBNext universal primer	AAT GAT ACG GCG ACC ACC GAG ATC TAC ACT CTT TCC CTA CAC GAC GCT CTT CCG ATC-s-T
NEBNext Index primer	CAAGCAGAAGACGGCATACGAGAT<u>NNNNNN</u>GTGACTGGAGTTCAGACGTGTGCTCTTCCGATC-s-T
	**For generation of the deep mutational scanning library**
For_B_NNS_HTH	GAGGTACAGTCCTATCTTGTGAGTGGAGAGCTGACAGCG<u>NNS</u>CATGCGCGTGCGATTGCCGCTGC
Rev_B_NNS_HTH	GTCCGGCAA<u>SNN</u>AAGAAGACGCAT<u>SNN</u>ATTCGCTACGTGAGA<u>SNN</u>ACTCTTGCCAATAGTTTGAGC
	**For the amplification of the pENTR backbone**
pENTR_gibson_backbone_R	ggtgaagggggcggccgcggagcctgc
pENTR_gibson_backbone_F	aagggtgggcgcgccgacccagctttcttg

Nucleotides in red font are spacer bases used to increase the diversity of the Illumina library. Underlined nucleotides are either Illumina library barcodes or NNS bases.

TABLE S4. X-RAY DATA COLLECTION AND PROCESSING STATISTICS. Related to Figures 2 and 4.

**Structure**	**ParB (DBD)-*parS* complex**	**Noc (DBD)-*NBS* complex**
Data collection		
Diamond Light Source beamline	I04	I03
Wavelength (Å)	0.980	0.976
Detector	Pilatus 6M-F	Eiger2 XE 16M
Resolution range (Å)	40.12 – 2.40 (2.49 – 2.40)	72.30 – 2.23 (2.66 – 2.23)[a]
Space Group	*C*2	*C2*
Cell parameters (Å/°)	*a* = 122.1, *b* = 40.7, *c* = 94.0, β = 121.4	*a* = 134.1 *b* = 60.6, *c* = 81.1, β = 116.9
Total no. of measured intensities	105021 (10942)	142152 (6183)
Unique reflections	16317 (1662)	10830 (542)
Multiplicity	6.4 (6.6)	13.1 (11.4)
Mean $I/\sigma(I)$	7.0 (2.0)	9.3 (1.5)
Completeness (spherical; %)	99.7 (99.2)	38.1 (4.7)
Completeness (ellipsoidal; %)	-	88.4 (57.2)
R_{merge}[b]	0.137 (0.801)	0.108 (0.851)
R_{meas}[c]	0.150 (0.869)	0.112 (0.891)
$CC_{½}$[d]	0.992 (0.850)	1.000 (0.847)
Wilson *B* value ($Å^2$)	42.1	115.7
Refinement		
Resolution range (Å)	40.12 – 2.40	72.30 – 2.23
Reflections: working/free[e]	15480/826	10231/599
R_{work}[f]	0.216	0.231
R_{free}[f]	0.232	0.279
Ramachandran plot: favoured/allowed/disallowed[g] (%)	96.5/3.5/0.0	95.4/4.6/0.0
R.m.s. bond distance deviation (Å)	0.003	0.002
R.m.s. bond angle deviation (°)	1.08	1.03

No. of protein residues per chain	121/140	105/116
No. of DNA bases per chain	20/20	22/22
No. of water/glycerol molecules	82/2	0/0
Mean *B* factors: protein/DNA/ water/overall (Å^2)	51/46/38/49	155/148/0/154
**PDB accession code**	**6S6H**	**6Y93**

Values in parentheses are for the outer resolution shell.

[a] After correction by STARANISO to remove poorly measured reflections affected by anisotropy, the ellipsoidal resolutions were:

2.23 Å in direction 0.854 a^* + 0.017 b^* - 0.519 c^*

3.83 Å in direction 0.302 a^* + 0.784 b^* + 0.543 c^*

4.02 Å in direction 0.130 a^* + 0.911 b^* + 0.391 c^*

[b] $R_{merge} = \sum_{hkl} \sum_i |I_i(hkl) - \langle I(hkl) \rangle| / \sum_{hkl} \sum_i I_i(hkl)$.

[c] $R_{meas} = \sum_{hkl} [N/(N-1)]^{1/2} \times \sum_i |I_i(hkl) - \langle I(hkl) \rangle| / \sum_{hkl} \sum_i I_i(hkl)$, where $I_i(hkl)$ is the ith observation of reflection *hkl*, $\langle I(hkl) \rangle$ is the weighted average intensity for all observations i of reflection *hkl* and N is the number of observations of reflection *hkl*.

[d] $CC_{½}$ is the correlation coefficient between symmetry equivalent intensities from random halves of the dataset.

[e] The dataset was split into "working" and "free" sets consisting of 95 and 5% of the data respectively. The free set was not used for refinement.

[f] The R-factors R_{work} and R_{free} are calculated as follows: $R = \sum(| F_{obs} - F_{calc} |)/\sum| F_{obs} |$, where F_{obs} and F_{calc} are the observed and calculated structure factor amplitudes, respectively.

[g] As calculated using MolProbity (Chen et al., 2010).

TABLE S5. DEEP MUTATIONAL SCAN AND ChIP-Seq. Related to STAR Methods.

**Deep mutational scanning libraries**	**GEO**
Pre-selection library, replicate 1	This study (GSE129285)
Pre-selection library, replicate 2	This study (GSE129285)
Pre-selection library, replicate 3	This study (GSE129285)
Post-selection library, selection for *parS*-binding capability, replicate 1	This study (GSE129285)
Post-selection library, selection for *parS* -binding capability, replicate 2	This study (GSE129285)
Post-selection library, selection for *parS* -binding capability, replicate 3	This study (GSE129285)
Post-selection library, selection for *NBS*-binding capability, replicate 1	This study (GSE129285)
Post-selection library, selection for *NBS* -binding capability, replicate 2	This study (GSE129285)
Post-selection library, selection for *NBS* -binding capability, replicate 3	This study (GSE129285)
**ChIP-seq datasets**	
TLE3000+pUT18C::1xFLAG-Bacillus subtilis Noc, fixation with 1% formaldehyde, α-FLAG antibody (Sigma), ChIP fraction	This study (GSE129285)
TLE3000+pUT18C::1xFLAG-Clostridium difficile Noc, fixation with 1% formaldehyde, α-FLAG antibody (Sigma), ChIP fraction	This study (GSE129285)
TLE3000+pUT18C::1xFLAG-Lactobacillus aviarius Noc, fixation with 1% formaldehyde, α-FLAG antibody (Sigma), ChIP fraction	This study (GSE129285)
TLE3000+pUT18C::1xFLAG-Staphylococcus aureus Noc, fixation with 1% formaldehyde, α-FLAG antibody (Sigma), ChIP fraction	This study (GSE129285)
TLE3000+pUT18C::1xFLAG-Bacillus subtilis ParB, fixation with 1% formaldehyde, α-FLAG antibody (Sigma), ChIP fraction	This study (GSE129285)
TLE3000+pUT18C::1xFLAG-Clostridium difficile ParB, fixation with 1% formaldehyde, α-FLAG antibody (Sigma), ChIP fraction	This study (GSE129285)
TLE3000+pUT18C::1xFLAG-Lactobacillus aviarius ParB, fixation with 1% formaldehyde, α-FLAG antibody (Sigma), ChIP fraction	This study (GSE129285)
TLE3000+pUT18C::1xFLAG-Staphylococcus aureus ParB, fixation with 1% formaldehyde, α-FLAG antibody (Sigma), ChIP fraction	This study (GSE129285)
TLE3000+pUT18C::1xFLAG-Caulobacter crescentus ParB, fixation with 1% formaldehyde, α-FLAG antibody (Sigma), ChIP fraction	This study (GSE129285)
TLE3000+pUT18C::1xFLAG-Agrobacterium tumefaciens ParB, fixation with 1% formaldehyde, α-FLAG antibody (Sigma), ChIP fraction	This study (GSE129285)
TLE3000+pUT18C::1xFLAG-Sinorhizobium meliloti ParB, fixation with 1% formaldehyde, α-FLAG antibody (Sigma), ChIP fraction	This study (GSE129285)
TLE3000+pUT18C::1xFLAG-Lawsonia intracellularis ParB, fixation with 1% formaldehyde, α-FLAG antibody (Sigma), ChIP fraction	This study (GSE129285)
TLE3000+pUT18C::1xFLAG-Desulfovibrio vulgaris ParB, fixation with 1% formaldehyde, α-FLAG antibody (Sigma), ChIP fraction	This study (GSE129285)
TLE3000+pUT18C::1xFLAG-Dechloromonas aromatica ParB, fixation with 1% formaldehyde, α-FLAG antibody (Sigma), ChIP fraction	This study (GSE129285)
TLE3000+pUT18C::1xFLAG-Pseudomonas aeruginosa ParB, fixation with 1% formaldehyde, α-FLAG antibody (Sigma), ChIP fraction	This study (GSE129285)
TLE3000+pUT18C::1xFLAG-Xanthomonas campestris ParB, fixation with 1% formaldehyde, α-FLAG antibody (Sigma), ChIP fraction	This study (GSE129285)
TLE3000+pUT18C::1xFLAG-Thermus thermophilus ParB, fixation with 1% formaldehyde, α-FLAG antibody (Sigma), ChIP fraction	This study (GSE129285)
TLE3000+pUT18C::1xFLAG-Bifidobacterium longum ParB, fixation with 1% formaldehyde, α-FLAG antibody (Sigma), ChIP fraction	This study (GSE129285)

TLE3000+pUT18C::1xFLAG-Mycobacterium tuberculosis ParB, fixation with 1% formaldehyde, α-FLAG antibody (Sigma), ChIP fraction	This study (GSE129285)
TLE3000+pUT18C::1xFLAG-Streptomyces coelicolor ParB, fixation with 1% formaldehyde, α-FLAG antibody (Sigma), ChIP fraction	This study (GSE129285)
TLE3000+pUT18C::1xFLAG-Porphyromonas gingivalis ParB, fixation with 1% formaldehyde, α-FLAG antibody (Sigma), ChIP fraction	This study (GSE129285)
